# Botanical Pesticides Against Fall Armyworm in African Maize Systems: A Structured Narrative Review and SWOT Synthesis

**DOI:** 10.3390/plants15111637

**Published:** 2026-05-27

**Authors:** Trust Kasambala Donga, Theresa Nakoma-Ngoma, Limbikani Matumba, James Bokosi

**Affiliations:** 1 Sustainable Crop Protection Group, Crop and Soil Sciences, Faculty of Agriculture, Bunda Campus, Lilongwe University of Agriculture and Natural Resources (LUANAR), Lilongwe P.O. Box 219, Malawi; 2 Foodplus Research Group, Department of Agriculture and Food Systems, NRC Campus, Lilongwe University of Agriculture and Natural Resources (LUANAR), Lilongwe P.O. Box 219, Malawi; 3 Plant Breeding Group, Crop and Soil Sciences, Faculty of Agriculture, Bunda Campus, Lilongwe University of Agriculture and Natural Resources (LUANAR), Lilongwe P.O. Box 219, Malawi

**Keywords:** plant-derived insecticides, agroecological pest control, bioinsecticides, pest suppression, farmer-based pest management

## Abstract

Maize (*Zea mays*) production in Africa has been severely constrained by the fall armyworm (FAW), *Spodoptera frugiperda*, since the pest was first detected on the continent in 2016. Botanical pesticides are increasingly promoted as low-cost alternatives to synthetic insecticides, but the African evidence base remains scattered across plant species, formulations, experimental settings, and outcome measures. This study was conducted as a structured narrative review with an evidence-informed SWOT (strengths, weaknesses, opportunities, and threats) synthesis to evaluate the efficacy, field relevance, and practical limitations of botanical pesticides used against FAW in African maize systems. Literature published between 2016 and 2026 was identified through structured searches of Google Scholar and PubMed and was synthesized narratively because substantial heterogeneity in botanicals, extraction methods, doses, application methods, target stages, and study settings precluded meta-analysis. The review shows that *Azadirachta indica* currently has the strongest balance of laboratory-to-field support, while *Tephrosia vogelii* and *Nicotiana tabacum* remain promising but with conditional options because of chemotype dependence and phytotoxicity concerns. Essential oils expand the mechanistic scope of the evidence base but are still limited by volatility, weak persistence, and comparatively few field validations in African maize systems. Overall, botanical pesticides have credible potential within smallholder-oriented integrated pest management, but require standardisation, better field validation and clearer biosafety evidence, and more accessible formulations are needed before broad recommendation can be justified.

## 1. Introduction

Fall armyworm (FAW), *Spodoptera frugiperda*, has become one of the most important invasive insect pests affecting maize production in Africa since its detection on the continent in 2016 [1]. Its rapid spread across diverse agroecological zones, broad host range, high reproductive capacity, and destructive feeding on maize have made it a major threat to food security and rural livelihoods (Supplementary Figure S1) [2,3]. The challenge posed by the fall armyworm is magnified by the fact that many maize-growing households operate under conditions of limited access to credit, extension support, irrigation, and purchased inputs [4,5]. Management systems in Africa have relied heavily on synthetic insecticides, largely because of farmer familiarity, and production of rapid visible effects. However, dependence on synthetic control has raised concerns related to affordability, pest resistance, farmer exposure, environmental contamination, and impacts on non-target organisms [6,7]. These concerns are especially important in smallholder systems, where safe use, correct timing, and sustained access to commercial pesticides cannot always be assumed. The biology of fall armyworm further complicates control. FAW larvae often become concealed within maize whorls, reducing the effectiveness of externally applied treatments [2,8]. These limitations have increased interest in integrated pest management approaches that combine different control options rather than relying on a single method [3,9]. Within this broader search for sustainable and locally adaptable alternatives, botanical pesticides have received increasing attention. Many pesticidal plants are already in farmer practice, are locally available, and can be prepared with limited cash expenditure [10,11]. Plant-derived products are also of scientific interest because they contain diverse bioactive compounds that may act through toxicity, repellence, feeding deterrence, or interference with insect development [6,12]. As a result, several botanicals, including neem (*Azadirachta indica*), fish bean (*Tephrosia vogelii*), tobacco (*Nicotiana tobacum*) and other plant species, have been evaluated for fall armyworm management under laboratory, screen/greenhouse, and field conditions in different African countries [10,13].

Despite this growing body of work, literature remains difficult to interpret in a clear, conclusive and comparative way. Studies differ widely in plant species, plant parts used, extraction methods, formulation types, concentrations, application methods, target larval stages, and response variables measured [14,15]. In many cases, efficacy is reported without sufficient attention to standardisation, persistence under field conditions, crop safety, labour requirements, or practical suitability for smallholder use [10,16]. This has made it difficult to judge not only whether botanical pesticides can suppress fall armyworm, but also how differences in preparation and use influence performance and what constraints limit wider application. A clearer synthesis is therefore needed to organize the evidence on botanical pesticides used against fall armyworm in African maize systems. In particular, the literature requires closer examination of the major botanicals studied, the ways in which plant materials are extracted, formulated, and applied, and the factors that influence efficacy under field conditions, including variability in performance, persistence, crop safety, biosafety, and practical usability. Against this background, the present paper examines the available evidence on botanical pesticides for fall armyworm management in Africa, with emphasis on the principal botanicals investigated, the methods used in their preparation and application, and the main constraints and opportunities affecting their field relevance.

## 2. Results and Discussion

### 2.1. Botanical Pesticides Evaluated Against Fall Armyworm in African Maize Systems

**Figure 1:**
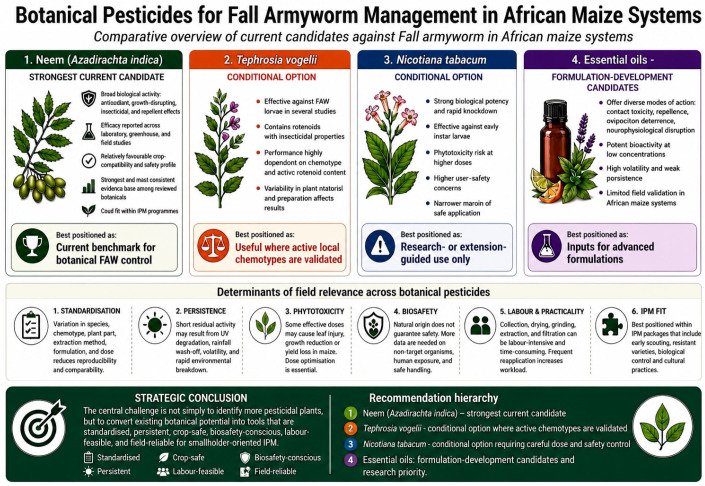
Comparative overview of botanical pesticide candidates and determinants of field relevance for fall armyworm management in African maize systems. Gemini AI free version.

The literature on botanical pesticides against fall armyworm (FAW), *Spodoptera frugiperda*, in African maize systems is large enough to permit a more discriminating synthesis than a simple species-by-species inventory. The main analytical problem is not only that different plant species have been tested, but that studies vary simultaneously in formulation type, plant part, extraction method, concentration, target pest stage, application route, and experimental setting [10,14,15]. As a result, apparent contradictions in efficacy often arise from unlike treatments being discussed as though they were directly comparable. The discussion is, therefore, organised first by formulation type and then by dominant bioactive class. To provide a visual synthesis of this evidence hierarchy, Figure 1 summarises the relative positioning of the main botanical pesticide candidates and the cross-cutting determinants that influence their field relevance in African maize systems.

#### 2.1.1. Crude Plant Extracts and Powders

Crude extracts and powders remain the most relevant formulation type for African maize systems as they align most closely with farmer practice and local plant availability with low cash investment [10,11]. These formulations are most vulnerable to inconsistency because the concentration of active compounds depend heavily on plant identity, plant part, freshness, extraction procedure, adjuvant use, and application timing [15,16]. The central question is not whether crude botanicals can affect the fall armyworm (FAW), but which of them currently have enough field-grounded evidence to justify cautious recommendation. A generalized extraction workflow for leaf-based botanical preparations is shown in Supplementary Figure S2.

##### Limonoid-Rich Botanicals

- *Azadirachta indica*

Among the crude botanicals evaluated against FAW in African maize systems, neem (*Azadirachta indica*) remains the best-supported candidate. Its relevance derives not simply from repeated reports of activity, but from the fact that neem has been tested across a wider range of African contexts and formulation types than most alternatives [6,10]. Azadirachtin and related limonoids act through growth disruption, antifeedant effects, and interference with development and reproduction, giving neem a broader biological signature than a purely acute toxicant [17,18,19,20]. This matters agronomically because reduction in feeding damage may occur even when mortality is incomplete or delayed [21,22,23]. Representative structures of azadirachtin and related active compounds discussed in this review are provided in Supplementary Figure S3 [17,24,25,26].

Across African studies, neem leaf extracts, powders, oils, and azadirachtin-based products have repeatedly reduced larval abundance, leaf damage, or both, and in several cases have protected grain yield relative to untreated controls [11,27,28,29,30,31,32,33,34,35]. The most useful recent studies are those that move beyond generic references to “neem extract” and specify dose and formulation. For example, 10% *w*/*v* aqueous leaf extracts performed well in Malawi when prepared with a surfactant [29]. A 2024 Ugandan study reported clear dose–response improvement with increasing powder concentration [31]. Subsequent field studies from Ethiopia and Zambia further support neem as a realistic FAW suppression option under smallholder conditions [28,35]. Soil-drench application of azadirachtin also suggests that the neem evidence base is beginning to diversify beyond simple foliar spraying [32].

Neem presently offers the strongest balance of evidence across biological activity, crop compatibility, and field relevance. Neem active ingredient content varies with plant source, plant part, post-harvest handling, and extraction method [17,20,29,30,31]. Azadirachtin is photosensitive, so efficacy commonly declines under strong sunlight, rainfall, and prolonged exposure [9,34]. Neem should be treated as the current benchmark for crude botanical control of FAW in African maize systems. Neem can also serve as the clearest example of how formulation determines whether botanical efficacy is reproducible.

##### Rotenoid-Rich Botanicals

Fish bean (*Tephrosia vogelii*) is clearly a biologically active pesticidal plant. Its efficacy depends fundamentally on phytochemical identity [24,36]. Visually similar plants may differ markedly in active ingredient concentration [36]. This chemotypic problem is central to interpreting the FAW evidence. The principal active constituents, especially deguelin and tephrosin, are rotenoids associated with disruption of mitochondrial respiration. Representative structures of the principal rotenoid compounds are shown in Supplementary Figure S3.

Positive results for *T. vogellii* have been reported under laboratory, semi-field, and farmer-linked conditions. Recent work shows that *Tephrosia*-containing treatments can reduce larval pressure and crop injury in maize [37,38,39]. However, contradictory outcomes in efficacy against the fall armyworm (FAW) are better explained by unverified chemotype and extraction quality than by simple experimental inconsistency [24,29,36,37]. Hence, *T. vogellii* is reliable only when the right chemistry is present.

Wild neorautanenia (*Neorautanenia mitis*) evidential status is more provisional. Its inclusion is justified because it reflects genuine farmer-linked and demonstration-based use in parts of southern Africa, particularly Malawi, rather than because it is already supported by a robust body of replicated FAW field trials [40,41]. The present evidence suggests practical visibility and plausible pesticidal activity, yet it does not provide the same analytical depth available for *A. indica*, tobacco (*Nicotiana tobacum)* or even *T. vogelii*.

The main limitation is not merely that there are few studies. It is that the available information does not yet resolve the most important practical questions: how reproducible efficacy is under maize field conditions, how safe the effective dose range is for the crop and the applicator, how long activity persists, and whether wider use would be ecologically sustainable. Because the tuber is commonly harvested, promotion of *N. mitis* raises a more serious sustainability issue than leaf-based botanicals [40,41]. For that reason, *N. mitis* should be retained as a locally important candidate but discussed explicitly as an under-validated one. At present, it identifies a relevant line of practice and research, not a defensible recommendation.

##### Alkaloid-Rich Botanicals

Tobacco, *Nicotiana tabacum*, is one of the most potent crude botanicals tested against the fall armyworm (FAW), and the literature is consistent in showing that nicotine-rich extracts can produce strong larval mortality under controlled conditions [11,14,29,38,39,42]. In mechanistic terms, tobacco differs from neem because its activity depends more heavily on rapid neurotoxic action than on growth regulation or feeding deterrence. This often makes tobacco appear superior in laboratory assays. The structure of nicotine as a representative alkaloid active ingredient is shown in Supplementary Figure S3.

Tobacco provides the clearest example of why biological potency cannot be equated with field suitability. Field work in Zambia and elsewhere suggests that tobacco extracts can reduce infestation and leaf damage [29,38,39,42]. Yet, the same evidence also shows a narrower safety margin than that for neem. Under field conditions, concentrations that suppress FAW can also inhibit maize growth or reduce yield [11].

In addition to phytotoxicity concerns, nicotine-rich preparations raise obvious risks, especially in low-input settings where protective equipment and dosing precision cannot be assumed [43]. Methodological inconsistency across studies further complicates interpretation because concentrations are reported in different units and preparation methods are poorly harmonized [39,42].

##### Other Crude Botanicals with Limited or Emerging Evidence

The remaining crude botanicals are discussed comparatively rather than as co-equal alternatives. Gliricidia (*Gliricidia sepium*) is currently the most credible profile in this group because it has been linked to both laboratory activity and positive field outcomes—reduced infestation and improved grain yield relative to untreated controls [44]. Chinaberry (*Melia azedarach*), Lemongrass (*Cymbopogon* spp.), basil (*Ocimum* spp.) and garlic (*Allium sativum*) also merit inclusion because field comparisons suggest agronomic promise, but those results remain difficult to generalise because formulations and concentrations are not yet standardised across studies [11,45,46,47]. By contrast, the evidence for *Jatropha* spp., *Piper nigrum*, and related taxa is still dominated by isolated or laboratory-heavy findings [48,49].

The critical point is that these species expand the candidate pool but do not yet alter the hierarchy of recommendation. Their importance is strategic rather than definitive: they may diversify locally available options, but the present evidence is too fragmented, geographically narrow, or methodologically inconsistent to place them at the same level as neem, or even as tobacco and *T. vogelii*. Treating them as a single emerging-evidence group, therefore, gives them appropriate visibility without inflating their recommendation status.

### 2.2. Essential Oils

Essential oils differ from crude extracts not only in chemistry, but also in the way they reach the insect, the speed with which they dissipate, and the kinds of formulation development requirements [50,51,52,53,54,55,56]. In most cases, they are rich in mono- and sesquiterpenes and act through a mixture of contact toxicity, fumigant action, repellence, feeding deterrence, and neurophysiological disruption. Their importance in this review lies less in immediate extension value than in showing that fall armyworm (FAW) is susceptible to a broad range of volatile plant compounds. A simplified illustration of essential-oil extraction by steam distillation is provided in Supplementary Figure S4.

#### 2.2.1. Monoterpene-Rich Oils

*Cymbopogon citratus*, *Ocimum* spp., and related oils.

The strongest case within this formulation type is for monoterpene-rich oils associated with *Cymbopogon*, *Ocimum*, and related aromatic genera. Lemongrass-type systems are dominated by citral-rich mixtures such as geranial and neral [56,57]. *Ocimum* and other Lamiaceae oils contain different monoterpene profiles that may nevertheless converge functionally in FAW suppression [52,53,54,58,59]. Across the broader literature, these oils have shown larvicidal, repellent, antifeedant, and enzymatic effects -such as acetylcholinesterase-related disruption and altered behavioural performance [51,52,53,54,60,61]. Representative terpenoid structures are shown in Supplementary Figure S5.

The main weakness of the essential-oil literature, especially from the standpoint of African maize systems, is the gap between laboratory activity and field persistence. Only a small subset of the evidence bears directly on field-relevant maize conditions, and even when activity is strong, volatility and environmental degradation tend to shorten residual effect [57,58,62,63]. Essential oils therefore currently function more convincingly as a formulation opportunity -how they might be stabilised and delivered, not simply in how strongly they kill larvae in closed assays [64,65].

#### 2.2.2. Other Volatile-Oil Systems with Emerging Evidence

Other volatile-oil systems, including those based on *Lippia*, *Mentha*, *Citrus*, *Piper*, and additional aromatic taxa, further broaden the chemical scope of the evidence base [49,51,65,66,67,68,69,70,71,72,73]. Some recent studies also report promising larvicidal or behavioural effects against the fall armyworm (FAW) [56,66,67]. However, this literature remains dominated by laboratory screening. As a result, these oils should be interpreted as an emerging research frontier rather than as evidence of field-ready farmer technologies [59,63,74].

### 2.3. Application Scope and Promotion Scenarios

For botanical pesticides, efficacy cannot be interpreted independently of where it is used. This is particularly true for fall armyworm (FAW) because the pest’s feeding behaviour, concealment within maize whorls, and overlap of egg, early-larval, and late-larval stages mean that timing and delivery route strongly influence apparent performance [9,10]. A treatment that is convincing in a laboratory bioassay may be operationally weak if it cannot reach the pest at the right stage under field conditions.

#### 2.3.1. Agroecological and Crop Context

The current evidence should be treated as agroecologically contingent rather than broadly transferable. Rainfall, irradiance, temperature, and crop growth rate affect both FAW pressure and the persistence of botanicals after application [2,10,63,75,76]. Consequently, African evidence from one agroecological zone should not be extrapolated uncritically to all maize-growing environments. The practical question is not whether a botanical works somewhere, but under what exposure conditions and management intensity it retains useful activity.

#### 2.3.2. Target FAW Developmental Stages and Timing of Control

Botanical treatments are more likely to be effective against eggs and early larval instars than against larger larvae already protected within the whorl [11,15]. This biological reality helps explain why laboratory efficacy often overestimates field performance: controlled assays usually guarantee exposure, whereas field outcomes depend on whether treatment reaches the pest before concealment reduces contact. Evidence on whorl application and early foliar treatment therefore deserves more interpretive weight than generic mortality figures alone [11,29,31].

#### 2.3.3. User Groups and Operational Scales

Recommendation strength also varies by user group. Farmer-prepared crude extracts may be feasible for low-input smallholders who have access to plant materials and can apply treatments on small areas [11,77]. The same options may be less realistic for larger-scale operations because preparation, filtration, and repeated application are labour-intensive. By contrast, essential oils and stabilised formulations may be more suitable for commercial product development or cooperative delivery than for household preparation. These distinctions should be made explicit because they determine whether a botanical belongs primarily in farmer practice, in extension-supported demonstration, or in product-development pipelines.

#### 2.3.4. Suitable Promotion Scenarios

Taken together, the evidence supports selective rather than blanket promotion. Neem (*A. indica*) has the strongest case for cautious extension-linked use where dose, preparation, and timing can be standardised [10,28,31,35]. *T. vogelii* may be useful where active chemotypes are known locally, but not where plant chemistry is uncertain [24,36,37,40]. Tobacco should only be promoted, if at all, under strongly qualified conditions because its efficacy is offset by crop-safety and user-safety concerns [11,43]. *N. mitis* and the lower-evidence crude botanicals remain better framed as locally relevant or research-linked options than as broad recommendations [40,41,45,48,49,78]. Essential oils, meanwhile, are best viewed as candidates for formulation development rather than as universally practical farmer-made interventions [61,74].

### 2.4. Cross-Cutting Determinants of Field Relevance

#### 2.4.1. Standardisation

Standardisation is the single most important condition for converting promising plant effects into defensible recommendation. Differences in plant part, chemotype, extraction ratio, surfactant use, soaking time, and reporting units make many studies loosely comparable [14,15,29,31]. The problem is especially severe for neem and *T. vogelii*, where apparently, similar treatments may in fact differ substantially in active composition [19,20,24,29,30,31,36]. Until protocols are standardised, contradictory findings will continue to reflect hidden treatment heterogeneity as much as genuine biological uncertainty.

#### 2.4.2. Persistence and Environmental Degradation

Persistence is the second major bottleneck. Neem degrades under light exposure, and essential oils dissipate rapidly through volatility and oxidation [10,34,63]. This is a direct explanation for why many botanicals look more convincing in laboratory or screenhouse settings than in open-field conditions. It also provides a clear bridge to opportunity: the growing literature on emulsions, nanoformulations, controlled-release systems, and other delivery technologies shows where future gains may arise [63,74,79]. A formulation innovation is only an agronomic opportunity if it remains accessible, affordable, and safe within African farming systems rather than merely improving laboratory performance.

#### 2.4.3. Phytotoxicity

Crop safety should be interpreted as part of efficacy, not as a separate afterthought. A treatment that suppresses FAW but depresses maize growth or yield has not solved the agronomic problem [11,51]. This point is most clearly illustrated by tobacco, but it extends more broadly to concentrated extracts and essential oils whose crop effects may depend on dose, carrier, and environmental conditions [11,51,80]. The literature would be much stronger if phytotoxicity were reported routinely alongside larval suppression, leaf damage, and yield.

#### 2.4.4. Non-Target Effects and Biosafety

The same principle applies to biosafety. Botanical origin does not automatically imply negligible ecological or occupational risk. Existing evidence suggests that many botanicals may reduce reliance on persistent synthetic insecticides, but evidence on parasitoids, predators, pollinators, soil organisms, and farmer exposure remains much less developed than evidence on target-pest toxicity [58]. This gap is especially relevant for concentrated oils, nicotine-rich extracts, and higher-potency formulations. Biosafety claims should therefore remain qualified unless they are supported by direct evidence rather than inferred from plant origin alone.

#### 2.4.5. Labour and Usability

Labour and usability are equally decisive. Farmer-made treatments often require collecting plant material, drying or pounding it, soaking and filtering extracts, and applying them repeatedly, sometimes directly into the whorl [11,31]. These requirements are not peripheral adoption issues; they are part of field relevance itself. A botanical that performs well experimentally but demands more labour than farmers can sustain during an outbreak may fail in practice even when the active compounds are effective.

#### 2.4.6. Fit Within IPM

For that reason, the most realistic role for botanical pesticides is within integrated pest management rather than as stand-alone substitutes for synthetic insecticides [10,16]. Their practical value is likely to be greatest when combined with scouting, early intervention, resistant germplasm, biological control, and other cultural or ecological measures [7,9]. The evidence reviewed here, therefore, supports a qualified but important conclusion: botanical pesticides are neither a marginal curiosity nor a universal replacement technology. They are an uneven but increasingly credible component of FAW management whose future depends less on discovering new plant species than on improving standardisation, formulation, timing, and fit with smallholder-oriented IPM systems.

To make the extracted evidence-base more transparent and directly comparable, Table 1 summarizes representative botanical–study combinations drawn from the core African maize evidence base discussed in Section 2.1, Section 2.2, Section 2.3 and Section 2.4. Research country and publication year are shown explicitly, and application rates are harmonized into directly comparable units, where reported. Values listed as NR were not reported in a directly comparable unit in the cited study or were not specified consistently enough in the manuscript to permit standardisation without overinterpretation.

### 2.5. Evidence-Based SWOT Synthesis of Botanical Pesticides Against Fall Armyworm in African Maize Systems

To ensure that the SWOT analysis is traceable to the reviewed literature, the synthesis in Table 2 is based only on evidence discussed in Section 2.1, Section 2.2, Section 2.3 and Section 2.4 and links each strategic point to the key supporting references. The table, therefore, serves not as a generic checklist, but as a citation-anchored synthesis of efficacy, field relevance, persistence, safety, usability, and promotion implications for African maize systems.

Taken together, the SWOT synthesis indicates that the main strategic limitation is not the absence of biologically active plants, but the weak translation of biological potential into standardized, persistent, crop-safe, labour-feasible, and field-reproducible practice. Accordingly, the most important opportunities lie in protocol standardisation, formulation improvement, field validation, biosafety assessment, and integration into smallholder-oriented IPM.

### 2.6. Conclusions

This structured narrative review shows that botanical pesticides have credible but uneven value for fall armyworm (FAW) management in African maize systems. The strongest current evidence supports neem-based treatments, which provide the most consistent balance of biological activity, field relevance, and crop compatibility across the reviewed African studies. By contrast, *Tephrosia vogelii* and *Nicotiana tabacum* remain biologically promising but more conditional options because their practical value depends more heavily on chemotype verification, crop-safety margins, handling constraints, and reproducible preparation protocols. Essential oils expand the mechanistic scope of FAW suppression, but their present limitation in African maize systems remains weak field persistence and comparatively thinner field validation.

Taken together, the evidence indicates that the main bottleneck is not the absence of active plant-derived products, but the weak conversion of botanical potential into standardized, field-reliable, labour-feasible, and biosafety-conscious practice. For this reason, the practical value of botanicals will depend less on expanding species lists than on improving standardisation of plant material and extraction procedures, defining crop-safe and effective doses, strengthening field validation, and developing formulations that improve persistence under African field conditions. The most defensible near-term role for botanical pesticides is therefore within smallholder-oriented integrated pest management (IPM), where standardized botanical use can complement scouting, early intervention, biological control, and other ecologically grounded tactics rather than be promoted as a universal stand-alone replacement for synthetic insecticides.

## 3. Materials and Methods

### 3.1. Review Design and Scope

This study was conducted as a structured narrative review with an evidence-informed SWOT synthesis focused on botanical pesticides against fall armyworm (FAW), *Spodoptera frugiperda*, in African maize systems. The review combined literature assembled during manuscript development with an updated verification search undertaken during revision. For structured evidence extraction, the core evidence base comprised primary studies that directly evaluated plant-derived interventions against FAW on maize under African conditions and reported extractable outcome data. Additional review articles and empirical studies were retained to support background framing and the interpretation of formulation, persistence, phytotoxicity, biosafety, non-target effects, and practical relevance for smallholder systems. Laboratory, screenhouse/greenhouse, on-station, and field studies were considered because the available African evidence base remains heterogeneous and relatively small.

### 3.2. Literature Search Strategy

An updated literature search was conducted on 15 April 2026 using Google Scholar and PubMed to verify the existing evidence base and identify more recent studies, particularly from 2024–2026. The search window covered January 2016 to April 2026 because FAW was first reported in Africa in 2016 [1] and the review was intended to capture evidence generated after the pest’s establishment on the continent. Search terms combined FAW identifiers with intervention, crop, and geographic terms. Representative search strings included: (“fall armyworm” OR “*Spodoptera frugiperda*” botanical pesticides, maize, Africa, neem, Tephrosia, tobacco, essential oils; (“*Spodoptera frugiperda*” AND maize AND Africa AND (botanical OR plant extract OR essential oil) AND (field OR screenhouse OR greenhouse)); (*Spodoptera frugiperda*) AND (botanical OR plant extract OR essential oil OR neem) AND (maize OR corn) AND Africa; and (“*Spodoptera frugiperda*” AND maize AND Africa AND (*Tephrosia vogelii* OR tobacco OR essential oil OR *Ocimum* OR *Cymbopogon*)). Search results were screened in ranked order on each platform.

### 3.3. Eligibility Criteria

Original research articles and review papers were eligible when they focused on botanical pesticides, plant extracts, pesticidal plants, essential oils, or related plant-derived products used against FAW in maize systems in African countries. Primary studies were included in the core extraction set when they reported direct evidence on efficacy, crop damage, yield response, phytotoxicity, persistence, or other information relevant to field interpretation and provided extractable data for African maize systems. Review papers were used for contextual and interpretive support but were not counted as core extractable studies. Publications were limited to English-language records. Conference abstracts, editorials, book chapters, non-African studies, studies unrelated to maize, and papers without a plant-derived intervention relevant to FAW management were excluded. Recent repository or preprint records were screened for contextual value but were used in the direct evidence base only when bibliographic details and study scope could be verified adequately.

### 3.4. Study Selection and Data Extraction

Titles and abstracts were screened by a single author (T.K.D.) for direct relevance to botanical management of fall armyworm (FAW) in African maize systems, and potentially eligible records were examined in greater detail at full-text or extended-record level. For the manuscript, 16 core primary studies directly matched the extraction frame of botanical efficacy against FAW in African maize systems and were used for structured evidence extraction. From these studies, the following information was extracted where available: botanical species, plant part, formulation, extraction method, concentration or dose, application method, frequency of application, target FAW stage, type of trial, country, study year, efficacy outcomes, leaf-damage response, grain yield, crop-safety observations, and any reported information on non-target or user safety. Additional empirical studies and review papers were used to support broader interpretation of mechanisms, formulation, persistence, biosafety, non-target effects, and IPM relevance, but were not counted in the core extraction set.

### 3.5. Evidence Classification and Synthesis

A quantitative meta-analysis was not attempted because the included studies differed substantially in botanical species, plant parts used, extraction procedures, formulations, concentration scales, target FAW developmental stages, exposure methods, response variables, and experimental settings. The evidence was therefore synthesised narratively. The synthesis proceeded at two levels: first, structured extraction and comparison of the 16 core African maize efficacy studies; and second, narrative integration of additional empirical and review sources used to interpret mechanisms, formulation issues, persistence, biosafety, and broader field relevance. To improve comparability, studies were interpreted by formulation type (for example, crude extracts and powders versus essential oils) and, where informative, by dominant bioactive class. The discussion also distinguished between laboratory potential and field relevance by separating laboratory, screenhouse/greenhouse, and field-oriented evidence. Recommendation strength was based on the overall weight of evidence, reproducibility across studies, crop compatibility, and operational relevance for African maize systems.

### 3.6. SWOT Framework Development

The SWOT analysis was developed from the extracted evidence rather than from an independent scoring exercise. Strengths were derived from repeatedly reported advantages such as biological activity against FAW, local availability of plant material, low cash cost, and compatibility with farmer-managed systems. Weaknesses were identified from recurrent limitations including inconsistent efficacy, weak standardisation, chemotype dependence, short residual activity, phytotoxicity risks, labour demands, and user-safety concerns. Opportunities were inferred from patterns in the literature pointing to formulation improvement, protocol standardisation, improved extension support, integration into integrated pest management (IPM), and the emergence of newer field studies from 2024–2026. Threats were derived from constraints such as poor reproducibility, uncertainty in plant material quality, ecological sustainability concerns, regulatory limitations, and the risk of overpromoting poorly validated interventions. The SWOT therefore represents an evidence-informed interpretive synthesis of the reviewed literature.

### 3.7. Methodological Limitations

This review has several limitations. The search relied on two platforms rather than a broader multi-database systematic-review workflow, the search was restricted to English-language records, and screening was conducted by a single author. In addition, the search used ranked retrieval rather than exhaustive database export and deduplication, and the available African evidence base remains heterogeneous in both design and reporting quality. For these reasons, the review is best interpreted as a structured narrative synthesis rather than as a formal systematic review or meta-analysis. Nevertheless, the updated search provides a transparent and current evidence base for evaluating botanical pesticides against fall armyworm (FAW) in African maize systems.

## Figures and Tables

**Table 1:** Comparative summary of representative extracted evidence on botanical pesticides against FAW in African maize systems (NR, not reported in a directly comparable unit).

Scientific Name of Botanical Pesticides/Essential Oil	Country	Pub. Year	Trial Setting	Formulation/ Plant Part	Standardized Rate/ Concentration	Main Reported Outcome	Ref.
*Azadirachta indica*	Malawi	2023	Field	Dry leaf powder (whorl)	3 g/whorl	Reduced larval abundance and leaf damage; yield protection reported	[11]
*Azadirachta indica*	Malawi	2020	Screenhouse/laboratory	Aqueous leaf extract	10% *w*/*v* (100 g/L)	Strong activity against early larvae; lower damage than control	[29]
*Azadirachta indica*	Benin	2021	Field	Neem oil	4.5 L/ha	Comparable or better field control than the semi-synthetic standard in some sites	[27]
*Azadirachta indica*	Uganda	2024	Field	Leaf powder/extract	200 g per L	Higher concentrations improved suppression under field conditions	[31]
*Azadirachta indica*	Zambia	2026	Field	Aqueous kernel extract	5% (*w*/*v*)/(50 g/L)	Effective suppression under smallholder field conditions reported	[28]
*Azadirachta indica*	Ethiopia	2025	Field	Aqueous kernel extract	600 g per L	Reduced FAW incidence and damage in small-scale farms	[35]
*Tephrosia vogellii*	Malawi	2020	Screenhouse/laboratory	Aqueous leaf extract	10% *w*/*v* (100 g/L)	Activity reported, but less consistent than neem under comparable tests	[29]
*Tephrosia vogelii* + *Azadirachta indica*	Uganda	2023	Greenhouse/field-linked	Combined leaf extracts	200 g/L	Combination treatment reported as promising against FAW	[37]
*Tephrosia vogellii*	Kenya	2023	Laboratory	Crude leaf extract	30 mg/L	Larvicidal activity reported against FAW	[39]
*Nicotiana tabacum*	Malawi	2023	Field	Dry leaf powder (whorl)	3 g/whorl	Strong suppression of larvae and damage, but agronomic caution required	[11]
*Nicotiana tabacum*	Zimbabwe	2020	Laboratory	Aqueous crude extract	25% (*w*/*v*)/175 g/L	Strong larvicidal activity reported on maize foliage diets	[42]
*Nicotiana tabacum*	Kenya	2023	Laboratory	Crude extract	30 mg/L	Activity reported against FAW alongside other crude botanicals	[39]
*Neorautanenia mitis*	Malawi	2024	Demonstration/field-linked	Tuber-based preparation	50 g/L	Locally important practice reported, but evidence remains under-validated	[40]
*Jatropha curcas*/neem oil systems	Chad	2022	Field	Oil formulations	1.25–1.4 L/ha	Botanical oils reported to reduce FAW under field conditions	[49]
*Lippia multiflora*/*Cymbopogon schoenanthus*	Burkina Faso	2024	Laboratory	Essential oils	NR	Repellent activity and adverse larval survival effects reported	[66]
*Ocimum gratissimum*, *Ocimum canum*, *Cymbopogon citratus*, *C. nardus* and *Citrus* sp.	Côte d’Ivoire	2023	Laboratory	Essential oils	NR	Larvicidal effects reported for several aromatic oil systems	[67]

**Table 2:** Citation-anchored SWOT synthesis of botanical pesticides against fall armyworm (FAW) in African maize systems.

SWOT Dimension	Evidence-Based Point	Strategic Implication	Key Supporting References
Strength	Many pesticidal plants are locally available and can be prepared with relatively low cash expenditure, which makes botanical control relevant to low-input smallholder systems.	Botanicals are most defensible where affordability, local access, and farmer-managed preparation are central to pest management decisions.	[10,76]
	Plant-derived products can affect FAW through multiple pathways: toxicity, repellence, feeding deterrence, and growth disruption, rather than through a single acute mechanism alone.	This broadens their value within integrated pest management and helps explain why damage reduction may occur even when rapid mortality is incomplete.	[6,12,17,18,19,20,50,51,52,53,55,63]
	Neem currently provides the strongest balance of laboratory, field, and agronomic support among the botanicals reviewed in African maize systems.	Neem is the clearest current benchmark for cautious recommendation and for comparing the practical readiness of other botanicals.	[11,27,28,29,30,31,32,33,34,35]
Weakness	Efficacy remains inconsistent because studies differ in plant species, plant part, extraction method, dose, surfactant use, and, in some cases, chemotype.	Standardisation and, where relevant, chemotype verification are needed before efficacy claims can be generalized with confidence.	[14,15,24,29,30,31,36]
	Residual activity is often short because of azadirachtin, and many volatile constituents degrade under sunlight, rainfall, heat, oxidation, or volatility.	Formulation improvement is needed to increase persistence and make field performance less dependent on immediate post-application conditions.	[10,34,46,63]
	Some otherwise promising botanical pesticides have narrower crop-safety or user-safety margins, with tobacco providing the clearest example of the efficacy-versus-safety trade-off.	Optimization and routine crop-safety reporting are essential before farmer-facing promotion can be justified.	[11,43,51,80]
	Labour demand remains high because farmer-made treatments often require collecting plant material, drying or pounding it, preparing extracts, filtering them, and applying them repeatedly.	Operational feasibility should be treated as part of efficacy assessment, especially in outbreak conditions and labour-constrained smallholder systems.	[11,31]
Weakness	Evidence on non-target effects, occupational exposure, and broader biosafety remains much thinner than evidence on target-pest suppression.	Biosafety claims should remain qualified until they are supported by direct evidence rather than inferred from plant origin alone.	[58,81]
Opportunity	The literature already points to tractable improvements through standardized preparation protocols, clearer dose reporting, and better source characterization of active plant material.	Research effort should prioritise improving reliability of the strongest candidates rather than simply expanding species lists.	[14,15,24,29,30,31,36]
	Emulsions, stabilized oils, encapsulation, and other delivery technologies could extend residual activity and improve leaf adhesion or dose consistency.	Short persistence can be treated as a formulation problem to be solved, not only as an intrinsic limitation of botanicals.	[63,74,79]
	Botanical pesticides are most likely to deliver value when embedded in IPM through early scouting, early-instar targeting, and integration with biological and cultural control.	The strongest promotion pathway is selective, context-specific, and IPM-linked rather than blanket substitution for synthetic insecticides.	[7,9,10,16]
Threat	Premature promotion of poorly validated botanicals, or of materials whose activity depends strongly on variable plant quality, chemotype, or destructive harvesting, could undermine farmer confidence and sustainability.	The main strategic threat is loss of credibility caused by overclaiming before interventions are sufficiently standardized, field-validated, and responsibly sourced.	[17,20,24,29,30,31,36,40,45,48,49,78]
	Several candidate botanicals remain under-validated, conditionally effective, or sustainability-constrained because efficacy may depend on variable plant quality or chemotype, evidence is still sparse, or use relies on destructive harvesting.	Overpromotion before these constraints is resolved risks presenting locally promising or research-stage botanicals as broadly field-ready recommendations.	[17,20,24,29,30,31,36,40,45,48,49,78]

## Data Availability

The original contributions presented in this study are included in the article/Supplementary Materials. Further inquiries can be directed to the corresponding author.

## Supplementary Materials

The following supporting information can be downloaded at: https://www.mdpi.com/article/10.3390/plants15111637/s1, Figure S1: Fall armyworm (*Spodoptera frugiperda*) larvae damage effects on foliar (A) and reproductive structures of maize (B). Photo credit: Trust Kasambala Donga. Figure S2: Flow diagram of extraction process of botanical pesticides used in management of fall armyworm (*Spodoptera frugiperda*) infestation in maize. Photo credit: Trust Kasambala Donga. Figure S3: Active ingredients, chemical structures and mode of action of major active ingredients found in selected botanical pesticides used to manage fall armyworm (*Spodoptera frugiperda*) infestation in Africa [17,24,25,26]. Figure S4: Illustration of essential oil extraction process using steam distillation. Figure S5: Active ingredients, chemical structures and mode of action of major active ingredients found in selected essential oils used to manage fall armyworm (*Spodoptera frugiperda*) infestation in Africa [56,62,63].

## Abbreviations

The following abbreviations are used in this manuscript:

FAW	Fall armyworm
SWOT	Strength, Weaknesses, Opportunities and Threats
