# Supplementary material for: Botanical Pesticides Against Fall Armyworm in African Maize Systems: A Structured Narrative Review and SWOT Synthesis
Source: Plants (Basel). 2026 May 27;15(11):1637. doi: 10.3390/plants15111637 (PMC13259547; doi:10.3390/plants15111637)

Figure S1: Fall armyworm larvae damage effects on foliar (A) and reproductive structures of maize (B). Photo credit: Trust Kasambala Donga.

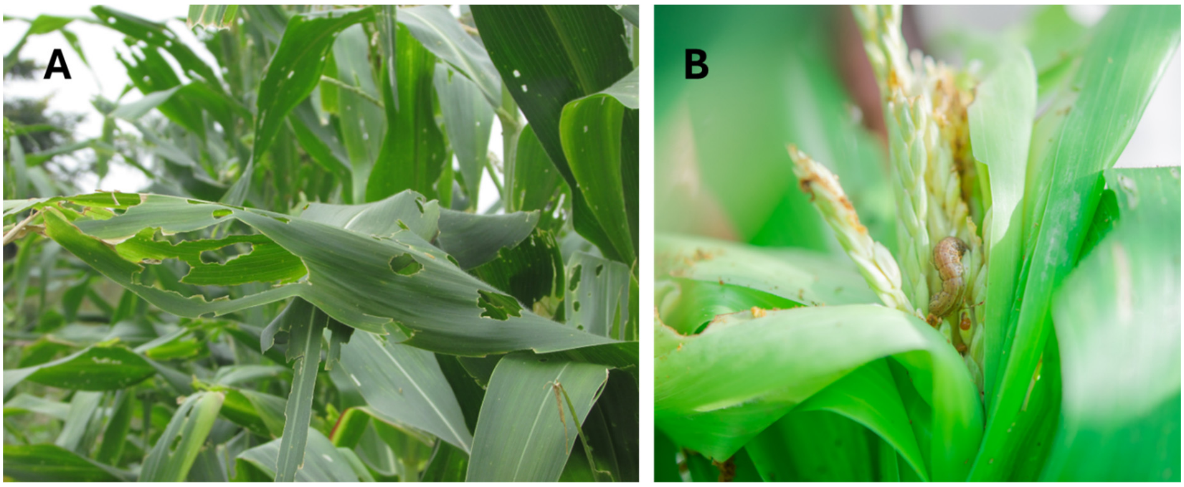

Figure S2: Flow diagram of extraction process of botanical pesticides used in management of fall armyworm infestation in maize. Photo credit: Trust Kasambala Donga.

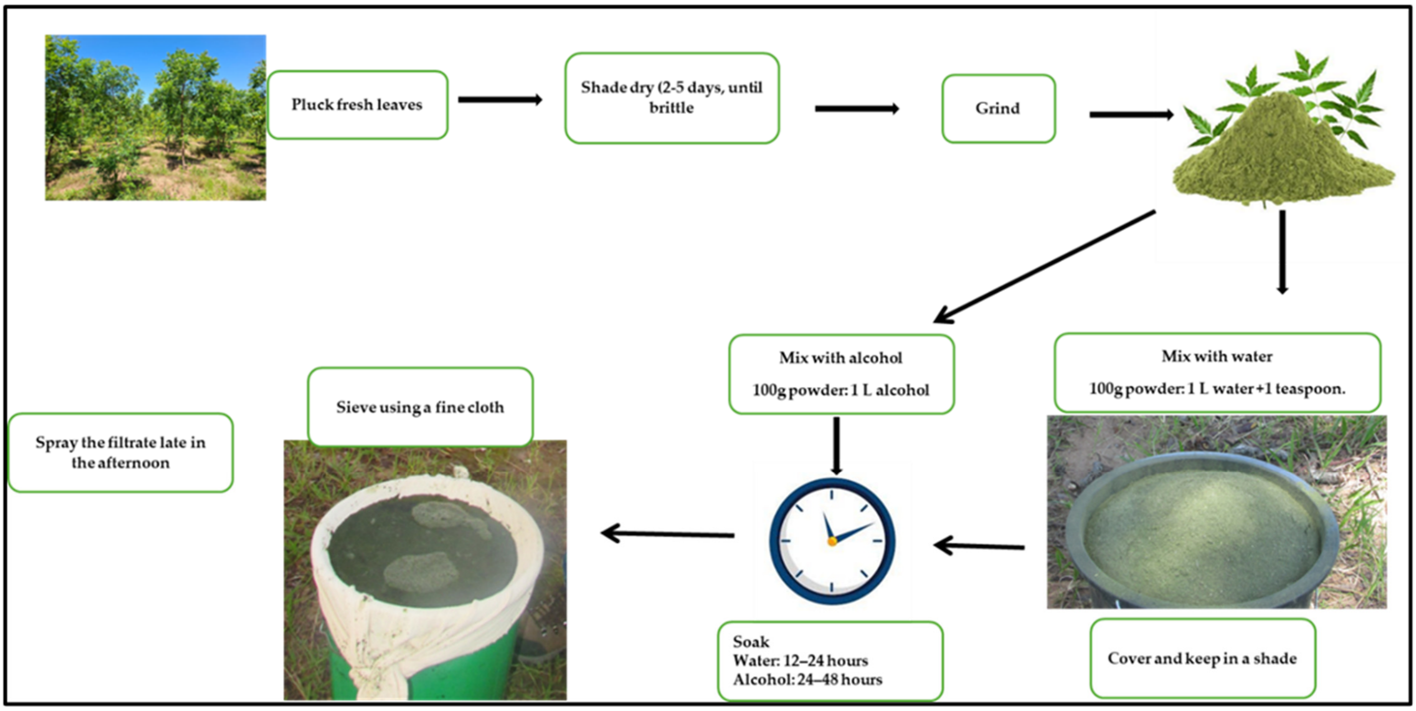

Figure S3: Active ingredients, chemical structures and mode of action of major active ingredients found in selected botanical pesticides used to manage fall armyworm infestation in Africa [17,24–26].

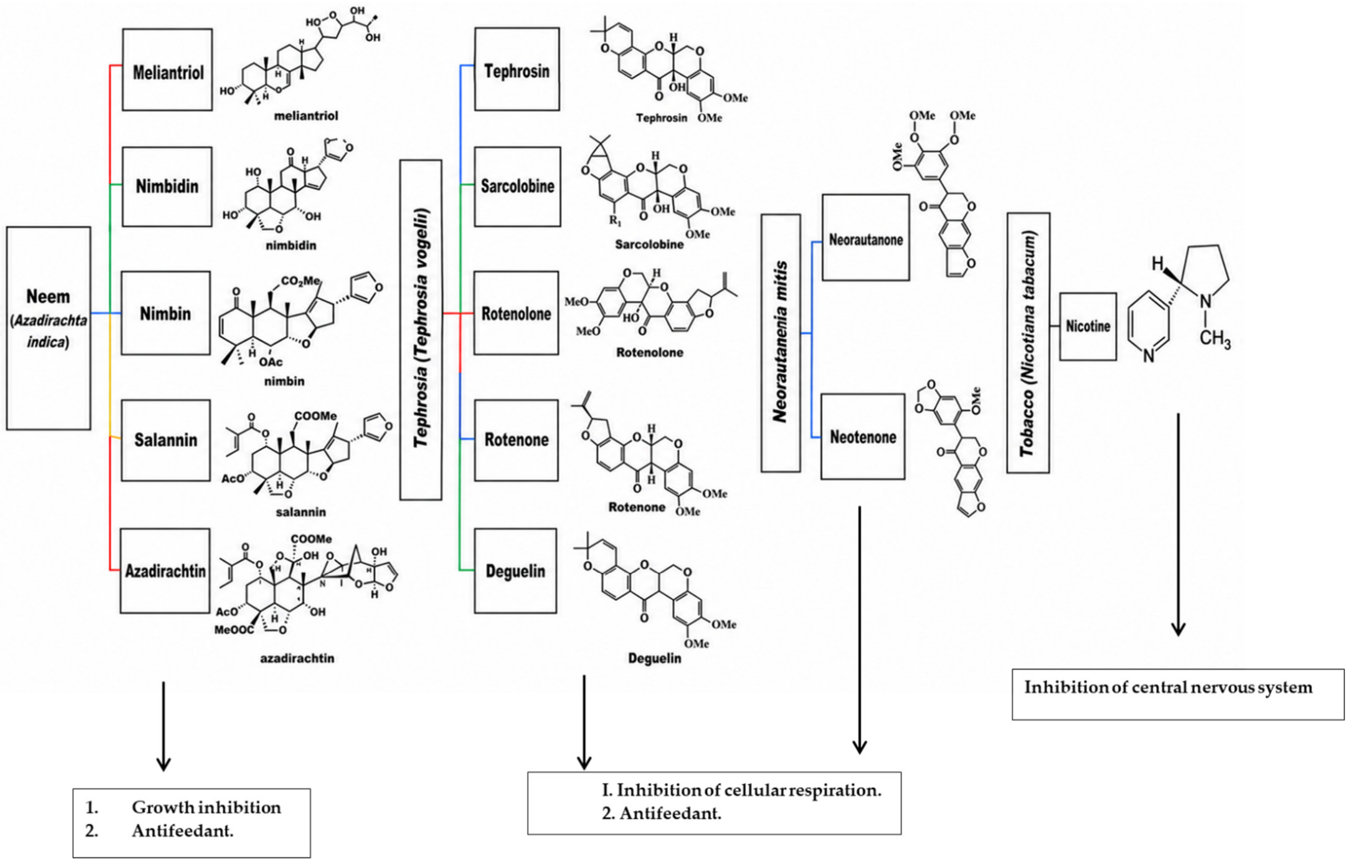

Figure S4: Illustration of essential oil extraction process using steam distillation

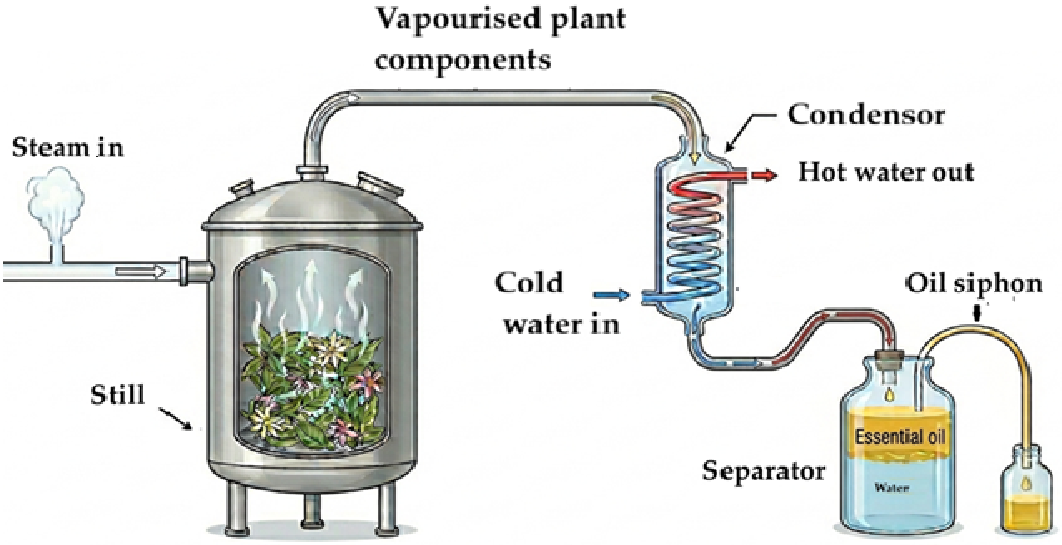

Figure S5: Active ingredients, chemical structures and mode of action of major active ingredients found in selected essential oils used to manage fall armyworm infestation in Africa [56,62,63].

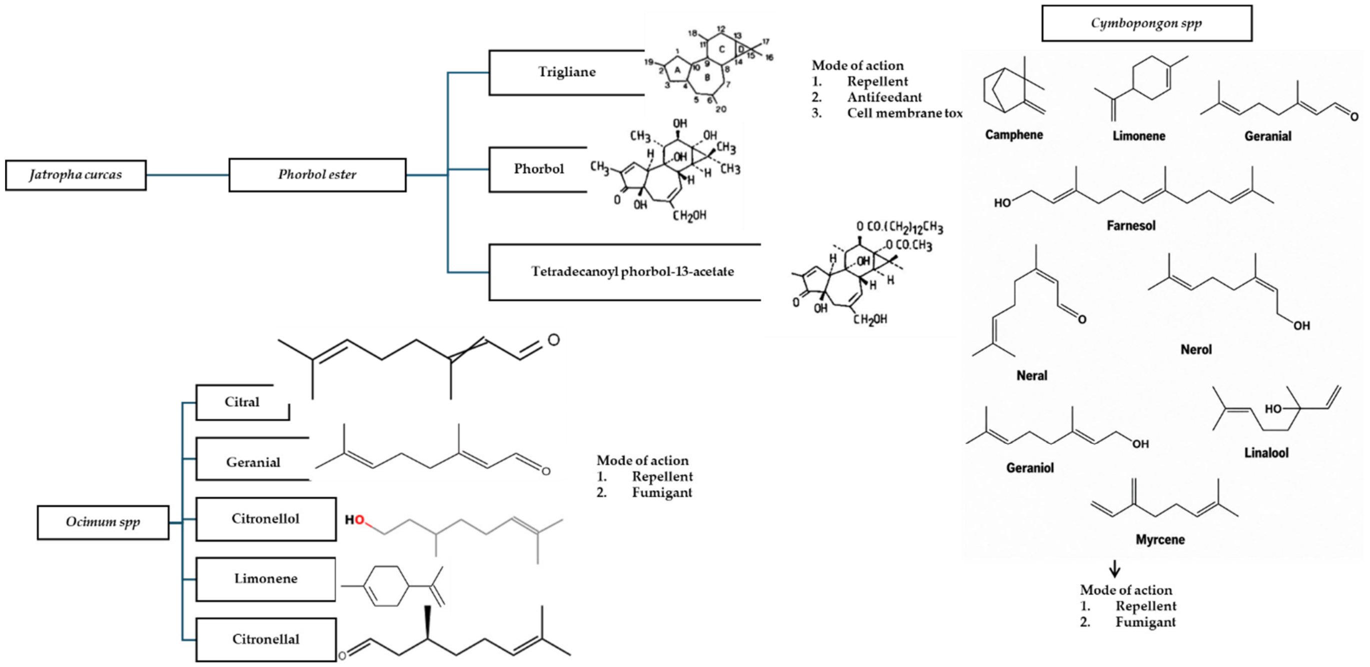

Supplement: Supplementary file 1 [file plants-15-01637-s001.zip › plants-4241441-supplementary.pdf]
